# Risk-Conferring Glutamatergic Genes and Brain Glutamate Plus Glutamine in Schizophrenia

**DOI:** 10.3389/fpsyt.2017.00079

**Published:** 2017-06-12

**Authors:** Juan R. Bustillo, Veena Patel, Thomas Jones, Rex Jung, Nattida Payaknait, Clifford Qualls, Jose M. Canive, Jingyu Liu, Nora Irma Perrone-Bizzozero, Vince D. Calhoun, Jessica A. Turner, Charles Gasparovic

**Affiliations:** ^1^Department of Psychiatry, University of New Mexico, Albuquerque, NM, United States; ^2^Department of Behavioral Sciences, University of New Mexico, Albuquerque, NM, United States; ^3^Department of Neurosciences, University of New Mexico, Albuquerque, NM, United States; ^4^Mind Research Network, Albuquerque, NM, United States; ^5^Department of Neurosurgery, University of New Mexico, Albuquerque, NM, United States; ^6^Department of Mathematics and Statistics, University of New Mexico, Albuquerque, NM, United States; ^7^The New Mexico VA Health Care System, Albuquerque, NM, United States; ^8^Department of Electrical Engineering, University of New Mexico, Albuquerque, NM, United States; ^9^Department of Psychology, Georgia State University, Atlanta, GA, United States

**Keywords:** glutamate, genetics, single-nucleotide polymorphisms, spectroscopy, schizophrenia

## Abstract

**Background:**

The proton magnetic resonance spectroscopy (^1^H-MRS) signals from glutamate (or the combined glutamate and glutamine signal—Glx) have been found to be greater in various brain regions in people with schizophrenia. Recently, the Psychiatric Genetics Consortium reported that several common single-nucleotide polymorphisms (SNPs) in glutamate-related genes confer increased risk of schizophrenia. Here, we examined the relationship between presence of these risk polymorphisms and brain Glx levels in schizophrenia.

**Methods:**

^1^H-MRS imaging data from an axial, supraventricular tissue slab were acquired in 56 schizophrenia patients and 67 healthy subjects. Glx was measured in gray matter (GM) and white matter (WM) regions. The genetic data included six polymorphisms genotyped across an Illumina 5M SNP array. Only three of six glutamate as well as calcium-related SNPs were available for examination. These included three glutamate-related polymorphisms (rs10520163 in *CLCN3*, rs12704290 in GRM3, and rs12325245 in *SLC38A7*), and three calcium signaling polymorphisms (rs1339227 in *RIMS1*, rs7893279 in *CACNB2*, and rs2007044 in *CACNA1C*). Summary risk scores for the three glutamate and the three calcium polymorphisms were calculated.

**Results:**

Glx levels in GM positively correlated with glutamate-related genetic risk score but only in younger (≤36 years) schizophrenia patients (*p* = 0.01). Glx levels did not correlate with calcium risk scores. Glx was higher in the schizophrenia group compared to levels in controls in GM and WM regardless of age (*p* < 0.001).

**Conclusion:**

Elevations in brain Glx are in part, related to common allelic variants of glutamate-related genes known to increase the risk for schizophrenia. Since the glutamate risk scores did not differ between groups, some other genetic or environmental factors likely interact with the variability in glutamate-related risk SNPs to contribute to an increase in brain Glx early in the illness.

## Introduction

Higher brain glutamate, glutamine, or glutamate plus glutamine (Glx) measured with proton magnetic resonance spectroscopy (^1^H-MRS) have been reported in schizophrenia, more consistently in subcortical regions (1). Though striatal elevations decrease with antipsychotic treatment (2), higher levels are reported in subjects at-risk for psychosis, as well as in never-medicated and chronic schizophrenia groups (1). This suggests that the elevations persist in medicated patients, although perhaps to a less severe extent and may be a trait variable (1). Consistent with this, in the largest sample to date (schizophrenia = 104, healthy control = 97), we reported higher Glx in medial frontal and parietal cortical regions, in clinically stable medicated schizophrenia patients (3). The *N*-methyl-d-aspartate receptor hypofunction model is a potential mechanism to account for increased glutamate metabolites in schizophrenia. It postulates dysfunction of these receptors in gamma-amino-butyric acid interneurons. Presumably, this results in disinhibition of pyramidal neurons and a paradoxical increase in presynaptic glutamate release across multiple cortical and subcortical fields. Though several genes have been proposed (4), the mechanisms accounting for increased glutamate in schizophrenia remain unknown.

Two studies have examined specific relationships of putative schizophrenia-related genes and brain glutamate levels in patients with the illness, with negative findings (5, 6). Recently, the Psychiatric Genetics Consortium [PGC (7)], the most comprehensive genome-wide association study to date, reported that 6 of 108 loci found to confer risk for schizophrenia involve genes clearly implicated in brain glutamate function (several other identified loci are likely to affect glutamate metabolism and synaptic function indirectly). However, there have been no investigations examining the impact of polymorphisms in these genes on brain glutamate concentrations in people with schizophrenia.

In the present study, we examined the relationships between three of the six glutamate-related risk-conferring single-nucleotide polymorphisms (SNPs) identified by the PGC and brain glutamate levels in schizophrenia and healthy control subjects (the other three SNPs could not be measured with the platform used). To evaluate the specificity of the relationship, we also examined correlations with calcium signaling SNPs also found to confer risk for schizophrenia by the PGC. A subgroup of subjects from our recent ^1^H-MRS study (3) for whom a saliva sample for genomics was collected was included. Because these common SNPs alleles clearly confer risk for the illness, are in genes directly involved in brain glutamate function (7), and Glx is abnormally increased in schizophrenia (1, 3), we hypothesized that schizophrenia subjects with a higher score of risk-conferring glutamate-related SNPs would have higher Glx brain levels. Because we previously reported increased Glx in both gray matter (GM) and white matter (WM) of medial frontal and parietal regions in schizophrenia subjects (3), we examined both tissue types without specific predictions.

## Materials and Methods

### Subjects

Patients with schizophrenia were recruited from the University of New Mexico Hospitals and the Albuquerque Veterans Affairs Medical Center. Inclusion criteria were (1) DSM IV TR diagnosis of schizophrenia made through consensus by two research psychiatrists using the information from a structured interview (SCID DSM IV TR, Patient Version), medical records, and family informants and (2) clinically stable on the same antipsychotic medications for at least 4 weeks. Exclusion criteria were (1) diagnosis of neurological disorder, (2) current substance use disorder (except for nicotine), (3) metallic implants, (4) claustrophobia, and (5) other than Caucasian ancestry. Healthy controls were additionally excluded for any of the following: (1) any current DSM IV TR axis I disorder [determined with SCID DSM IV TR Non-Patient Version; (except current nicotine) or any past history of a disorder (except for substance use)] and (2) a first degree relative with a psychotic disorder. The local internal review board approved the study. Subjects provided written informed consent and were paid for their participation.

### Clinical Assessments

Patients were assessed for psychopathology with the Positive and Negative Syndrome Scale (8), the Simpson Angus Scale [SAS (9)] for parkinsonism, the Barnes Akathisia Rating Scale (10), and the Abnormal Involuntary Movement Scale (11). Assessments were completed within 1 week of scanning.

### Magnetic Resonance Studies

#### Acquisition

**Figure 1:**
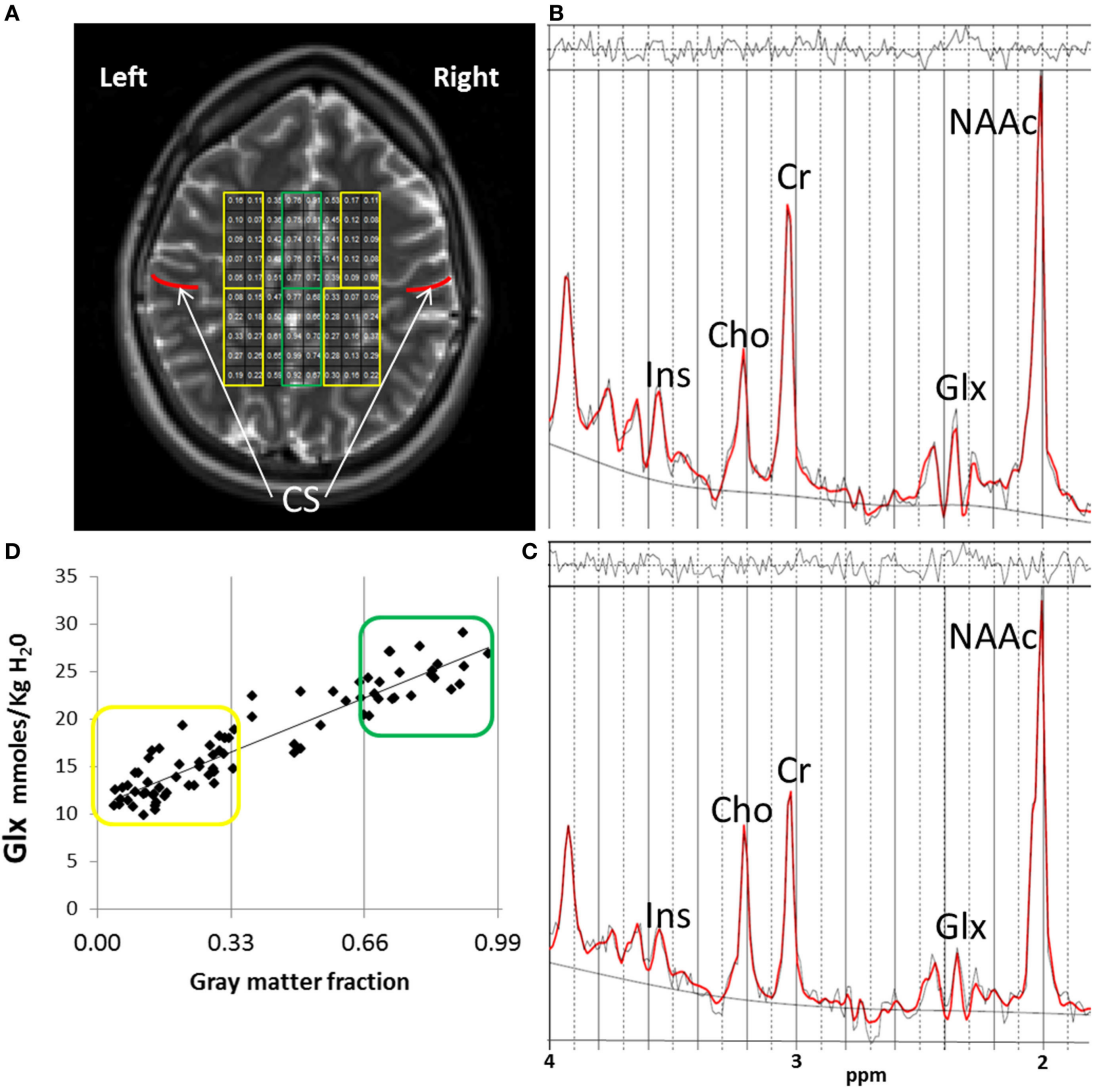
^1^H-MRSI methods. **(A)** ^1^H-MRSI axial supraventricular slab placement with highlighted predominantly white matter (WM) (in yellow) and gray matter (GM) (in green) voxels. Although a field-of-view of 32 × 32 is acquired, the volume of interest analyzed in all subjects and presented here, is a rectangular subset that fits within the oval of the brain, minus the most outer rows and columns to reduce chemical shift voxel displacement artifact. Regions anterior to the central sulcus (CS, in red) are frontal. Regions posterior to the CS are parietal. **(B)** Example of one fitted spectrum (red line) from a predominantly GM voxel. Peak areas for glutamate + glutamine (Glx), *N*-acetyl-aspartate compounds (NAAc), total-creatine (Cr = Phosphocreatine + Creatine), myo-inositol (Ins), and total-choline (Cho = glycerophosphocholine + phosphocholine) are labeled. Top irregular line represents the residual signal after fitting. Lower continuous line represents the baseline used for fitting with LC Model. **(C)** Example of one fitted spectrum from a predominantly WM voxel. **(D)** Distribution of Glx values corresponding to the individual voxel’s GM fraction (GM/GM + WM) for the ^1^H-MRSI axial supraventricular slab from **(A)**. In yellow are predominantly WM and in green predominantly GM Glx values.

Scanning was performed on a 3-T scanner (VB-17; 12 channel head-coil). Subjects were told to try to remain awake during the acquisition but no task was implemented. T1-weighted images were collected with 3D-MPRAGE for voxel tissue segmentation (TR/TE/TI 1,500/3.87/700 ms, flip angle 10°, field-of-view = 256 mm × 256 mm, 1-mm thick slice). ^1^H-MRS imaging was performed with a phase-encoded version of a point-resolved spectroscopy sequence, to allow the simultaneous acquisition of multiple voxels as described previously (12). Acquisitions were obtained both with and without water presaturation. The following parameters were used: TE = 40 ms, TR = 1,500 ms, slice thickness = 15 mm, FOV = 220 mm × 220 mm, circular *k*-space sampling (radius = 12), Cartesian *k*-space size = 32 × 32 after zero filling, *k*-space Hamming filter with 50% width and number of averages = 1, total scan time = 582 s. The nominal voxel size was 0.71 cm^3^ but the effective voxel volume is estimated to be 2.4 cm^3^, taking into account the *k*-space sampling and filtering. The ^1^H-MRSI volume of interest was prescribed from an axial T_2_-weighted image to lie immediately above the lateral ventricles and parallel to the anterior–posterior commissure axis, and included portions of the cingulate gyrus and the medial frontal and parietal lobes (Figure 1A). To minimize the chemical shift artifact, the transmitter was set to the frequency of the NAA methyl-peak during the acquisition of the metabolite spectra and to the frequency of the water-peak during the acquisition of the unsuppressed water spectra.

#### Spectral Fitting

Data were automatically preprocessed and fitted using LC Model [Version 6.1 (13)] with a simulated basis-set for the sequence parameters which included the following metabolites: alanine, aspartate, creatine, phosphocreatine, gamma-amino-butyric acid, glutamine, glutamate, glycerophosphocholine, phosphocholine, myo-inositol, lactate, *N*-acetyl-aspartate, *N*-acetyl-aspartylglutamate, scyllo-inositol, and guanidine. Lipids and macromolecule contributions were fitted using the default simulated intensities of LC Model, which included soft constraints for peak position and line width and prior probabilities of the ratios of macromolecule and lipid peaks. Spectra were fitted in the range between 1.8 and 4.2 ppm and scaled to the non-suppressed water intensity (12). The SD of the fit of the Glx signals, provided by LC Model (related to the Cramer–Rao lower bounds and a measure of the confidence of the fit) was used to exclude data with low confidence. Only data with an LC Model SD ≤ 20% were further analyzed (13) (Figures 1B,C).

#### Partial-Volume–Relaxation Correction

The Glx results from LC Model were corrected as described previously and are reported in units of molality (moles/kg tissue water) (14). Briefly, the Glx signals were corrected for partial-volume and relaxation effects using GM, WM, and cerebrospinal fluid (CSF) maps from segmented T1-weighted images with SPM5. Water densities and relaxation times in each tissue or CSF compartment were obtained from the literature, for this correction (14). Our group has previously documented the test–retest reliability of these methods (12). As previously discussed (3), voxels were further classified based on their GM fraction as [100 × GM/(GM + WM) as “predominantly” GM (>66%) or “predominantly” WM < 34%; Figure 1D]. Finally, because glutamate and glutamine concentrations vary depending on tissue type and most voxels contained various proportions of GM and WM (15), we also used the GM fraction of each voxel as a covariate in the statistical analyzes (see below).

### Genetics

#### SNP Selection and Analyzes

Our aim was to study the six SNPs identified in the PGC (7) involved in glutamatergic function and the six SNPs involved in calcium signaling (Table 1). The genetic data originated from each subject’s saliva samples processed *via* 5M Illumina HumanOmni5-Quad SNP array (Illumina, www.illumina.com) and we used Illumina Genome Studio Genotyping Module to optimize call rates. Genome wide scan data from each participant underwent quality assurance testing before inclusion in the analysis. Requirements per SNP included a minor allele frequency greater than 5%, Hardy–Weinberg equilibrium (*p* < 10^−6^), and data for over 90% of participants in the sample. Three individuals missing over 10% of the total SNPs found in the platform were excluded. Only three of the glutamate-related and three of the calcium signaling SNPs were available for 125 subjects.

**Table 1:** Genotype distribution of the available glutamate-related and calcium signaling risk single-nucleotide polymorphisms (SNPs) from the PGC study in schizophrenia and healthy control subjects.

Schizophrenia PGC				Genotype distribution (current sample)						
	Gene	SNP	Rank (of 108)	Available	Homozygous non-risk		Heterozygous		Homozygous risk	
					*HC*	*Sz*	*HC*	*Sz*	*HC*	*Sz*
Glutamate	*GRM3* (7q21.12)	rs12704290	48	Yes	0.0	0.02	0.13	0.1	0.86	0.88
	*CLCN3* (4q.33)	rs10520163	59	Yes	0.16	0.29	0.59	0.55	0.23	0.16
	*SLC38A7* (16q21)	rs12325245	98	Yes	0.84	0.83	0.16	0.14	0.0	0.03
	*GRIN2A* (16p13.2)	rs9922678	90	No	–	–	–	–	–	–
	*GRIA1* (5q33.2)	rs79212538	79	No	–	–	–	–	–	–
	*SRR* (17p13.3)	rs4523957	47	No	–	–	–	–	–	–

Calcium	*CACNA1C* (12)	rs2007044	4	Yes	0.52	0.45	0.31	0.40	0.16	0.16
	*CACNB2* (10)	rs7893279	20	Yes	0.21	0.22	0.16	0.17	0.63	0.60
	RIMS1 (6q12-13)	rs1339227	108	Yes	0.05	0.07	0.27	0.24	0.69	0.69
	*CACNA1* (22q13.1)	Chr22_39987017_D	41	No	–	–	–	–	–	–
	*NRG* (11)	rs55661361	24	No	–	–	–	–	–	–
	*ATP2A2* (12)	rs4766428	58	No	–	–	–	–	–	–

*PGC is Psychiatric Genetics Consortium (7); HC is healthy control group (*n* = 67); Sz is schizophrenia group (*n* = 58)*.

#### Imputation

Any missing genotypes at the six loci of interest were selectively imputed via IMPUTE2 software (v2.3.1) and the 1000 Genomes Phase 3 reference dataset. SNPs rs12704290, rs12325245, and rs1339227 were imputed for all subjects. SNP rs2007044 was imputed for 1 subject. Imputed SNPs demonstrated a 0.9 or greater imputation probability estimate.

#### Adjustment

For each subject, the number of risk alleles (1 for homozygous non-risk, 2 for heterozygous, and 3 for homozygous risk) was multiplied by the odds ratio (OD) for schizophrenia from the PGC (12) at each SNP. In order to examine the potential effect of ethnicity, we used PLINK to calculate 10 multidimensional scaling (MDS) factors (equivalent to principal components) from the subjects’ SNP array data. The top principal components are generally associated with population structure, in other words ethnicity information. The top MDS factors were entered into the relevant PROC-MIXED model.

#### Summary Scores

Each SNP OD-adjusted risk value was summed to a total risk score (range 3–9) for glutamate-related SNPs and for calcium signaling SNPs. The direction of effect on Glx brain levels of a risk allele is unknown. However, as proof of concept, the total risk score approach supports the testing of the hypothesis that a higher risk score would positively correlate with the more abnormal (higher) Glx levels in schizophrenia [reported in the literature (1, 3)].

### Statistical Analysis

We examined whether the relationships between brain Glx and glutamate-related and calcium signaling genetic risk scores differed across schizophrenia and healthy controls. Because Glx brain levels differ by tissue type and age (15), and there are progressive tissue changes in schizophrenia (16), these variables (age and voxel tissue composition) must be considered in the analyzes. PROC-MIXED (SAS version-9) uses all available data, accounts for correlation between repeated measurements in the same subject (i.e., Glx in many voxels), and can handle missing data more appropriately than other methods (17). Hence, we implemented *four* repeated-measures PROC-MIXED analyzes: glutamate-related risk score and Glx in GM (1) and in WM (2); as well as calcium signaling risk score and Glx in GM (3) and in WM (4) (*p* = 0.05/4, Bonferroni-corrected *p* = 0.0125). Each of these omnibus tests included *Glx* concentration in all selected voxels as the repeated-measures dependent variable, with the following independent variables: *risk score* as the within-subject factor, *diagnostic-group* (schizophrenia, healthy control) as the between-group factor and age as a covariate. In order to facilitate the visualization (by plotting data) of the hypothesized correlations between Glx concentrations (continuous) and risk scores (continuous), age was dichotomized into *age-group* for the model; we chose a median split of ≤36 years as a neutral cutoff (results did not differ with age dichotomization of <30 or >45).

As in our previous ^1^H-MRS imaging study (3), we followed a hierarchical, systematic approach to statistical analyzes. To address type-1 errors, only the highest order significant interactions involving *diagnostic-group* and *risk score* (the relevant variables of interest) are presented in the *Results* and followed-up with PROC-MIXED *post hoc* tests; this protects the *post hoc* tests for type-I errors (18, 19). In order to control for effects of *diagnostic-group* differences in spectral fitting [e.g., bias in metabolite values due to worse quality in the ill group as previously reported (3)], we used the Cramer–Rao lower bounds (CRLB = SD/concentration × 100). The CRLB (not the SD reported by LC Model) should be used to account for group differences in spectral quality, since CRLB is independent of concentration (personal communication, Provencher, creator of LC Model). Hence, if the groups differed in CRLBs, GM fraction, relevant demographic or substance use characteristics, these were entered into the model as additional covariates. The potential confound of antipsychotic medication was examined by adding the drug dosage as standardized olanzapine equivalents (20) to the appropriate model in the schizophrenia group. Likewise, the effects of various symptom severity and neurological side-effects measures were examined in each relevant model. All tests were two-tailed and we used Satterthwaite’s correction for unequal variances.

## Results

### Demographics, Substance Use History, and Quality of Spectral Fitting

Fifty-eight schizophrenia and 67 healthy controls were studied. There were no significant differences between the groups in: age, gender, Hispanic ethnicity, socioeconomic status (SES) of the subject, or SES of the family of origin, smoking status, vascular risk factors, or history of opiate or sedative use disorders (Table 2). Schizophrenia subjects had more frequent lifetime histories of alcohol (*p* = 0.006), cannabis (*p* < 0.001), cocaine (*p* = 0.02), hallucinogens (*p* = 0.004), and stimulant use disorders (*p* = 0.05). Also, the schizophrenia group had slightly, but significantly, higher CRLB for Glx (Glx_CRLB_) in GM (*F*_1,123_ = 53.3, *p* < 0.001) and WM (*F*_1,123_ = 36.9, *p* < 0.001). However, schizophrenia and controls did not differ in GM fractions for GM (*p* = 0.11) or for WM (*p* = 0.15). As expected, CSF proportion across voxels was higher in the schizophrenia than the control group (*p* < 0.001). This difference was addressed by the partial-volume correction method (14).

**Table 2:** Demographic, clinical, and spectral quality characteristics of the subjects.

	Schizophrenia (*n* = 58)	Healthy controls (*n* = 67)
Age, years	38 ± 14	36 ± 12
Gender (male/female)	46/12	49/18
Hispanic (yes/no)	19/37	24/43
Socioeconomic status (SES)	4.5 ± 1.6	4.3 ± 1.5
Parental SES	4.1 ± 1.7	3.9 ± 1.5
Smoker (yes/no)	13/42	18/49
Vascular risk score^a^	2.0 ± 1.8	2.3 ± 1.8
History of alcohol use disorder (yes/no)	18/36	7/54*
History of cannabis use disorder (yes/no)	15/39	1/60*
History of hallucinogen use disorder (yes/no)	4/50	0/61*
History of stimulant use disorder (yes/no)	7/47	0/61*
History of cocaine use disorder (yes/no)	5/49	0/61*
History of opiate use disorder (yes/no)	3/51	0/61
History of sedative use disorder (yes/no)	2/52	0/61
Current smoker (yes/no)	13/42	18/49
Glx_CRLB_^b^ gray matter (GM)	3.8 ± 0.8	3.6 ± 0.7*
Glx_CRLB_ white matter (WM)	2.6 ± 0.6	2.5 ± 0.4*
GM fraction GM	0.819 ± 0.1	0.813 ± 0.1
GM fraction WM	0.150 ± 0.08	0.146 ± 0.08
Cerebrospinal fluid per voxel	0.085 ± 0.1	0.073 ± 0.09*
Age onset psychosis	20.9 ± 8.4	N/A
Positive symptoms	14.8 ± 5.2	N/A
Negative symptoms	14.6 ± 4.0	N/A
Tardive dyskinesia	4.2 ± 3.6	N/A
Parkinsonism	9.5 ± 2.0	N/A
Akathisia	0.2 ± 0.5	N/A
Antipsychotic dose in mgs^c^	14.8 ± 12.5	N/A

*p < 0.05.

*^a^Vascular risk score, 0–4 (score of 1 each for cardiac illness, hypertension, dyslipidemia, and diabetes)*.

*^b^CRLB is Cramer–Rao lower bounds (CRLB = SD × metabolite concentration/100). Glx is glutamate + glutamine*.

*^c^In olanzapine equivalents (20)*.

*± is for SD*.

### Glx and Glutamate-Related Risk Scores

#### Gray Matter

**Figure 2:**
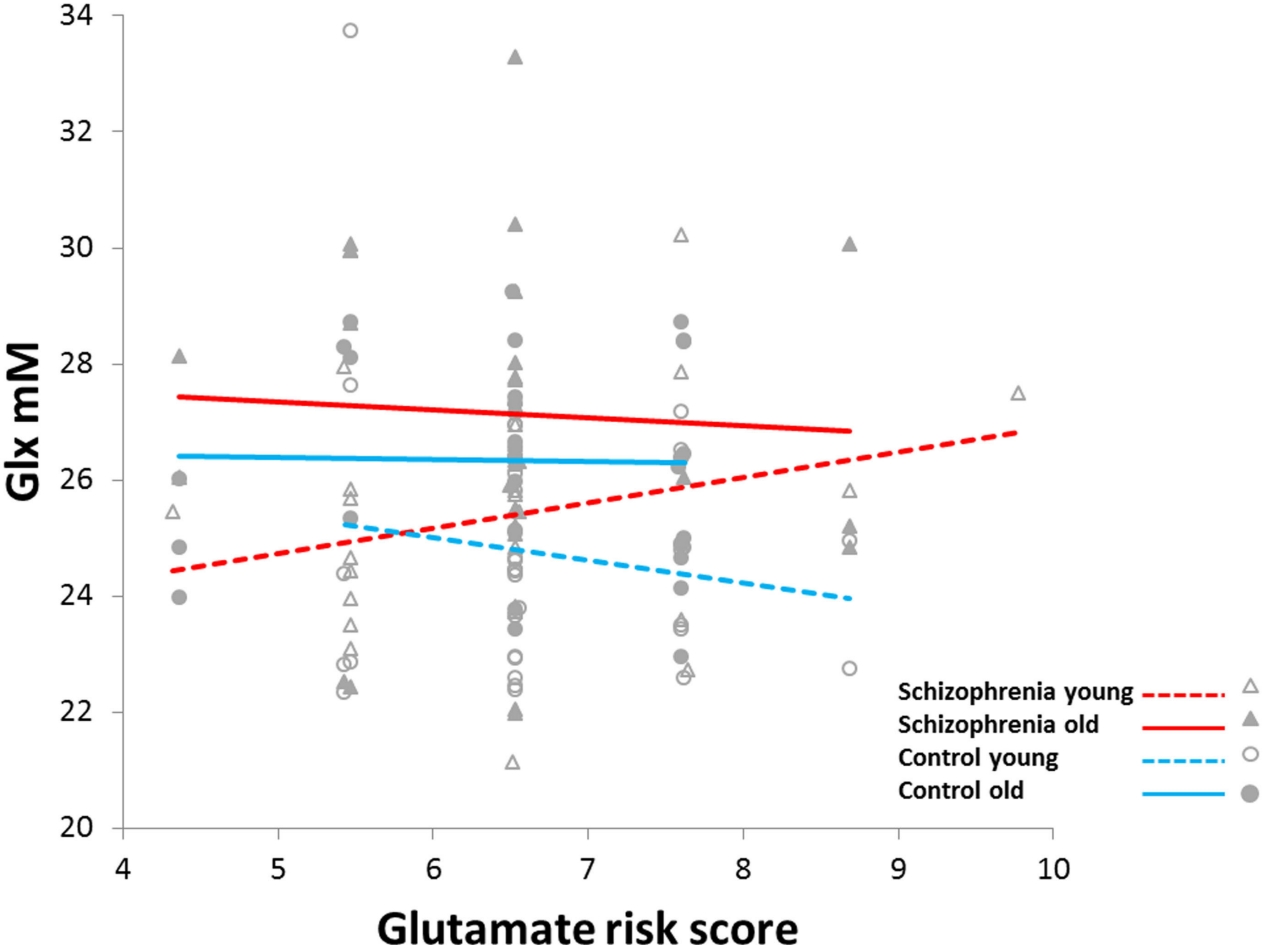
Increased slope of glutamine plus glutamate (Glx) concentration versus glutamate-related risk score in younger schizophrenia (<36 years) compared to younger controls, older schizophrenia (≥36 years), and older controls (*F*_1,117_ = 6.8, *p* = 0.01).

Glx was positively correlated with glutamate risk score but only in the younger schizophrenia group (*diagnostic-group* × *age-group* × *risk-score*: *F*_1,117_ = 6.8, *p* = 0.01; Figure 2). This three-way interaction remained after adjusting for Glx_CRLB_ (actually became more robust, *F*_1,117_ = 10.5, *p* = 0.001), as well as after controlling for histories of alcohol, cannabis, cocaine, stimulant, and hallucinogen use (*p*’s between 0.002 and 0.003). Also the three-way interaction remained after adding the top 3, 4, or 5 MDS factors to address potential effects of ethnicity (*p*’s between 0.02 and 0.008). *Post hoc* PROC-MIXED confirmed that among the younger *age-group*, the relationships between Glx and *risk-score* differed between the schizophrenia and control groups (*F*_1,59_ = 9.9, *p* = 0.003). However, among the older *age-group*, these correlations did not differ between schizophrenia and control groups (*F*_1,58_ = 0.55, *p* = 0.46). Still, when examining the whole schizophrenia group, the associations between Glx and risk score differed between the younger and the older *age-groups* (*F*_1,54_ = 5.9, *p* = 0.02). Finally, in a test randomizing the dependent variable (Glx) 1,000 times, the observed (6.8) or higher *F* values occurred in 2 of the 1,000 permutations (*p* = 0.002). This provides further support that the above findings are unlikely to be the result of chance variations.

The differences in relationships between Glx and genetic score among the younger and older schizophrenia *age-groups* remained when accounting for age of onset of psychosis (*p* = 0.04), positive (*p* = 0.04) and depressive symptoms (*p* = 0.03), antipsychotic dose (*p* = 0.01) and severity of tardive dyskinesia (*p* = 0.007), parkinsonism (*p* = 0.02), and akathisia (*p* = 0.02). However, the differences became a trend when adjusting for negative symptoms score (*F*_1,52_ = 3.5, *p* = 0.07). Finally, severity of negative symptoms did not differ between younger (14.9 ± 4.3) and older (14.2 ± 3.8) schizophrenia patients (*p* = 0.5).

#### White Matter

A significant *diagnostic-group* × *age-group* × *risk-score* interaction (*F*_1,117_ = 7.7, *p* = 0.007), disappeared when adjusting for Glx_CRLB_ (*F*_1,117_ = 0.2, *p* = 0.65). There were no other significant interactions or main effects involving *diagnostic-group*; see Supplementary Material for full statistical model.

### Glx and Calcium Signaling Risk Scores

#### Gray Matter

The only significant effect involving diagnosis was the *diagnostic-group* × *risk-score* interaction (*F*_1,119_ = 5.0, *p* = 0.03). However, significance disappeared when adjusting for Glx_CRLB_ (*F*_1,119_ = 0.05, *p* = 0.82).

#### White Matter

A significant *diagnostic-group* × *age-group* × *risk-score* interaction (*F*_1,117_ = 8.4, *p* = 0.005), disappeared when adjusting for Glx_CRLB_ (*F*_1,117_ = 0.2, *p* = 0.63).

### Group Differences for Genetic Risk Scores and Glx Levels

Not surprisingly in this small sample, genetic *risk-scores* did not differ between the schizophrenia and the healthy controls for glutamate (*t*_123_ = 1.02, *p* = 0.31) or for calcium-related (*t*_123_ = 0.02, *p* = 0.98) SNPs. Glx was higher in the schizophrenia compared to the control group, adjusting for age or tissue type (*F*_1,122_ = 26.5, *p* < 0.001); however, this is not a new finding but merely a statement that the subgroup examined in this report behaves similar to the full sample from the original study (3) in terms of Glx levels.

## Discussion

We found that among younger subjects with schizophrenia, scores in glutamate-related risk-conferring SNPs positively correlated with Glx levels in GM. In older schizophrenia patients, as in the healthy controls regardless of age, there was no such relationship. This pattern of relationships was not found for *risk-scores* in neuronal calcium signaling SNPs and was not accounted by variance in antipsychotic dose or other common confounds, such as prior substance use histories or the quality of spectral fitting. Finally, Glx levels were higher in the schizophrenia group but the genetic risks scores did not differ from the healthy controls.

Only two other studies have examined the relationship between a glutamate-related gene and *in vivo* brain glutamate in schizophrenia. Ongur et al. (5) reported that a haplotype of four SNPs within the glutaminase 1 (GLS1) gene was positively associated with the glutamine/glutamate ratio (Gln/Glu) in the parieto-occipital cortex. The sample included a combination of subjects with schizophrenia, bipolar disorder, and healthy controls but there was no difference in the haplotype score versus Gln/Glu correlation across the groups. Gruber et al. (6) found that methionine homozygous carriers for the val66met SNP (*rs6265*) of the brain-derived neurotrophic factor (BDNF) gene had lower hippocampal glutamate in a combined group of schizophrenia, bipolar disorder, and healthy controls. Again, there were no differences in the associations across groups. However, the PGC did not rank any SNPs in GLS1 or BDNF into the top 108 schizophrenia risk loci (7).

What do these findings tell us about the pathophysiology of schizophrenia? The positive correlation between glutamate-related genes and GM Glx in the younger schizophrenia group is somewhat specific (i.e., not seen with the calcium signaling genes). *GRM3* codes for a glutamate receptor predominantly expressed in astrocytes (21); *CLCN3* is a voltage-gated chloride channel critical for glutamate reuptake in synaptic vesicles of neurons (22); and *SLC38A7* encodes a sodium-coupled l-glutamine transporter expressed in neurons (23). In addition, ^1^H-MRS visible Glx includes metabolic and neurotransmitter glutamate pools, as well as glutamine, and most glutamine is the product of synaptic glutamate re-uptaked by glial cells (24). Hence, variability in specific common SNPs in these genes, known to confer risk for schizophrenia, can be plausibly related to abnormally increased levels of Glx in GM, though elucidation of the specific mechanisms will require additional experimental approaches. However, the normal range in glutamate *risk-scores* found in our schizophrenia sample is not sufficient to account for increases in Glx. Hence, some other factors, genetic and/or environmental, must interact with the glutamate genetic risk to increase glutamatergic cortical levels during the early course of the illness. Likewise, this relationship is not apparent in older schizophrenia subjects, with similar glutamate risks scores, also suggests that other factors affect Glx concentration in schizophrenia. Epigenetic factors, like differential methylation of glutamate risk genes during the course of illness, could potentially affect Glx brain levels. In support of this possibility, our recent MRI/genetic/epigenetic preliminary data showed that variation in methylation of PGC gene loci is more robustly related to GM concentration reductions in schizophrenia than the variability in the risk-conferring SNPs themselves (25). Alternatively, other non-specific factors like aging or disease duration may increase Glx levels and obscure a relationship with risk scores in the older subjects.

This study had several strengths, including the assessment of many GM and WM voxels with standardized metrics of quality of spectral fits. As in our recent report (3), controlling for group differences in CRLB can have major effects on the results. However, some limitations must be acknowledged. First, the sample size is small, and replication is necessary. We are not currently aware of a similar ^1^H-MRS imaging database of supraventricular Glx in schizophrenia with broad SNP characterization for a replication. However, ours is the first proof-of-principle study documenting an association between risk-conferring glutamate-related SNPs and Glx brain levels in schizophrenia. With greater standardization of ^1^H-MRS protocols, future larger studies would be able to clearly document the extent of specific genetic contributions to glutamatergic dysfunction. Second, glutamate was not resolved from glutamine in this study, and Glx levels do not reflect the rate of glutamatergic metabolism, which would be a more functionally relevant measure. Glutamate and glutamine are present in all brain cell types, so ^1^H-MRS measurements combine several functional compartments. Hence, interpretation of Glx brain differences is not straight forward. ^13^C-MRS, though technically demanding and yet to be widely applied in large human samples, could in future studies assess more directly glutamate and glutamine metabolic cycling and their relationship to glutamate-related risk genes in schizophrenia. Third, Glx measurements were acquired without controlling for cognitive state, which can affect glutamate levels (26). Fourth, schizophrenia subjects were treated with antipsychotic medications, agents known to affect brain glutamate levels (2). However, adjustment for antipsychotic dosage within the schizophrenia group, did not cancel the difference in correlations between *risk-score* and Glx concentrations across the younger and older *age-groups*. Fourth, only three of the six glutamate and three of the six calcium-related SNPs from PGC were examined due to limited coverage of the Illumina SNP array used. Hence, a complete assessment of the six risk-conferring SNPs could yield different results. Furthermore, it is possible that other risk genes involved in a metabolic pathway that feeds into glutamatergic neurotransmission may also be related to Glx brain concentrations. Fifth, the cortical regions studied were not found to have increased Glx in a recent meta-analysis (1). However, the meta-analysis was published before our recent study, which has by far the largest sample [*N* = 201 (3)]; still the supplemental data of the meta-analysis showed a small effect size (0.12) for Glx greater in Sz than controls in medial frontal cortex, consistent with our results. Sixth, our criteria for excluding subjects with missing SNP data (>10% of the total SNPs) may be somewhat liberal. Finally, the schizophrenia group had a greater past history of several substance use disorders that could affect Glx levels (27). However, controlling for this history did not eliminate the main findings.

In summary we report in younger schizophrenia patients, a positive relationship between GM Glx levels, with a combined score for glutamate-related SNPs found to confer risk for the illness. This relationship is somewhat specific, as it is not present in WM (which also had increased Glx in schizophrenia), or for calcium signaling SNPs (which also confer risk for schizophrenia). The overall findings suggest that though variance in some common SNPs may indeed contribute to the increased cortical glutamate levels in schizophrenia, other genetic and/or environmental mechanisms must also be involved early in the disease. Future studies very early in the illness with greater Glx brain coverage and examining epigenetic factors that modulate the impact of specific risk-conferring SNPs may shed further light on the underlying neurobiology of glutamatergic dysfunction in schizophrenia. Also, modulators of presynaptic glutamate release may be particularly effective for patient subgroups early in the illness and which have dysregulation of central nervous system glutamatergic tone (28).

## Ethics Statement

This study was carried out in accordance with the recommendations of UNM-HSC Human Research Review Committee with written informed consent from all subjects. All subjects gave written informed consent in accordance with the Declaration of Helsinki. The protocol was approved by the Human Research Review Committee.

## Author Contributions

JB: design, data collection and analyses, and writing of the manuscript. VP, NP, TJ, RJ, CQ, NP-B, JL, JC, JT, VC, and CG: data analyses and writing of the manuscript.

## Conflict of Interest Statement

JB received honoraria for advisory board consulting from Otsuka America Pharmaceutical Inc. in 2013. VP, TJ, RJ, NP, CQ, JC, JL, NP-B, VC, JT, and CG reported no biomedical financial interests or potential conflicts of interest.
