# Supplementary material for: Risk-Conferring Glutamatergic Genes and Brain Glutamate Plus Glutamine in Schizophrenia
Source: Front Psychiatry. 2017 Jun 12;8:79. doi: 10.3389/fpsyt.2017.00079 (PMC5466972; doi:10.3389/fpsyt.2017.00079)
Supplement: Supplementary file 1 [file data_sheet_1.docx]

**SUPPLEMENT TEXT**

**A) PROC MIXED analyses**. Below we present the best full model for four repeated-measures PROC MIXED (SAS version-9) analyses (p=0.05/4, Bonferroni-corrected p=0.0125): glutamate related risk score and Glx in gray (1) and in white matter (2), as well as calcium signaling risk score and Glx in gray (3) and in white matter (4). Each of these omnibus tests, has Glx concentration in all selected voxels as the repeated measures dependent variable, with the following independent variables: risk score as the within subject factor, diagnostic group (schizophrenia , healthy control) as the between group factor and age as a co-variate (with a median split of ≤36 years). Glx_CRLB_ was added as a co-covariate.

*Glutamate plus glutamine (Glx) and glutamate related risk scores*

1) Gray matter.

diagnosis x risk score x age (F_1,117_=6.8, p=0.01). Adjusting for Glx_CRLB_  (F_1,117_=10.5, p=0.002)

diagnosis x risk score (F_1,117_=4.2, p=0.04)

diagnosis x age (F_1,117_=9.2, p=0.003)

risk score x age (F_1,117_=0.3, p=0.6)

age (F_1,117_=3.4, p=0.07)

risk score (F_1,117_=0.2, p=0.7)

diagnosis (F_1,117_=2.7, p=0.1)

2) White matter. (adjusting for Glx_CRLB_)

diagnosis x risk score x age (F_1,117_=0.2, p=0.7).

diagnosis x risk score (F_1,117_=0.2, p=0.6)

diagnosis x age (F_1,117_=0.15, p=0.7)

risk score x age (F_1,117_=0.2, p=0.65)

age (F_1,117_=0.06, p=0.8)

risk score (F_1,117_=2.3, p=0.13)

diagnosis (F_1,117_=0.18, p=0.7)

*Glutamate plus glutamine (Glx) and calcium signaling risk scores*

3) Gray matter. (adjusting for Glx_CRLB_; diagnosis x risk score x age *and* risk score x age were non-significant).

diagnosis x risk score (F_1,119_=0.05, p=0.82)

risk score x age (F_1,119_=3.99, p=0.05)

age (F_1,119_=2.8, p=0.1)

risk score (F_1,119_=1.21, p=0.27)

diagnosis (F_1,119_=0.25, p=0.62)

4) White matter. (adjusting for Glx_CRLB_)

diagnosis x risk score x age (F_1,117_=0.23, p=0.63).

diagnosis x risk score (F_1,117_=1.2, p=0.27)

diagnosis x age (F_1,117_=0.3, p=0.58)

risk score x age (F_1,117_=4.5, p=0.03)

age (F_1,117_=1.6, p=0.2)

risk score (F_1,117_=6.7, p=0.01)

diagnosis (F_1,117_=1.4, p=0.24)
